# Trends in the Baidu Index in Search Activity Related to Mpox at Geographical and Economic Levels and Associated Factors in China: National Longitudinal Analysis

**DOI:** 10.2196/44031

**Published:** 2023-08-23

**Authors:** Min Du, Wenxin Yan, Lin Zhu, Wannian Liang, Min Liu, Jue Liu

**Affiliations:** 1 Department of Epidemiology and Biostatistics School of Public Health Peking University Beijing China; 2 Center for Primary Care and Outcomes Research, School of Medicine, Center for Health Policy Freeman Spogli Institute for International Studies Stanford University Stanford, CA United States; 3 Vanke School of Public Health Tsinghua University Beijing China; 4 Institute for Global Health and Development Peking University Beijing China; 5 Global Health and Infectious Diseases Group Global Center for Infectious Disease and Policy Research Peking University Beijing China; 6 Key Laboratory of Reproductive Health National Health and Family Planning Commission of the People’s Republic of China Beijing China

**Keywords:** mpox, internet attention, emergency, disparities, China

## Abstract

**Background:**

Research assessing trends in online search activity related to mpox in China is scarce.

**Objective:**

We aimed to provide evidence for an overview of online information searching during an infectious disease outbreak by analyzing trends in online search activity related to mpox at geographical and economic levels in China and explore influencing factors.

**Methods:**

We used the Baidu index to present online search activity related to mpox from May 19 to September 19, 2022. Segmented interrupted time-series analysis was used to estimate trends in online search activity. Factors influencing these trends were analyzed using a general linear regression (GLM) model. We calculated the concentration index to measure economic-related inequality in online search activity and related trends.

**Results:**

Online search activity was highest on the day the first imported case of mpox appeared in Chongqing compared to 3 other cutoff time points. After the day of the first imported mpox case in Taiwan, the declaration of a public health emergency of international concern, the first imported mpox case in Hong Kong, and the first imported mpox case in Chongqing, national online search activity increased by 0.642%, 1.035%, 1.199%, and 2.023%, respectively. The eastern regions had higher increases than the central and western regions. Across 31 provinces, municipalities, and autonomous regions, the top 3 areas with higher increases were Beijing, Shanghai, and Tianjin at 3 time points, with the exception of the day of the first imported mpox case in Chongqing (Chongqing replaced Tianjin on that day). When AIDS incidence increased by 1 per 100,000 people, there was an increase after the day of the first imported mpox case in Chongqing of 36.22% (95% CI 3.29%-69.15%; *P*=.04) after controlling for other covariates. Online search activity (concentration index=0.18; *P*<.001) was more concentrated among populations with a higher economic status. Unlike the central area, the eastern (concentration index=0.234; *P*<.001) and western areas (concentration index=0.047; *P*=.04) had significant economic-related disparities (*P* for difference <.001) in online search activity. The overall concentration index of changes in online search activity became lower over time.

**Conclusions:**

Regions with a higher economic level showed more interest in mpox, especially Beijing and Shanghai. After the day of the first imported mpox case in Chongqing, changes in online search activity were affected by AIDS incidence rate. Economic-related disparities in changes in online search activity became lower over time. It would be desirable to construct a reliable information source in regions with a higher economic level and higher AIDS incidence rate and promote public knowledge in regions with a lower economic level in China, especially after important public events.

## Introduction

Recently, a multiple-country mpox outbreak has attracted public attention. Beginning with the first human mpox case from the Democratic Republic of the Congo in 1970, sporadic zoonosis caused by *Orthopoxvirus*, namely, the Central African clade (clade I) and the West African clade (clade II), began to spread in rural rainforest villages of western and central Africa [1,2]. Prior to May 2022, human mpox was mainly epidemic in African countries [3]. However, since the United Kingdom reported a confirmed mpox case in a returnee from Nigeria, subsequent mpox virus infections occurred in multiple nonepidemic countries [4,5]. As of July 26, 2023, a total of 88,549 confirmed mpox cases across 113 countries or territories were reported globally [6].

The World Health Organization (WHO) International Health Regulations Emergency Committee officially announced that the mpox epidemic constituted a public health emergency of international concern (PHEIC) on July 23, 2022 [7]. With the development of the global economy, infectious disease outbreaks usually result in a public reaction in numerous countries other than the outbreak country. Although up to October 30, 2022, there were no local mpox cases in mainland China, imported cases were identified in Taiwan, Hong Kong, and Chongqing on June 24, September 6, and September 16, 2022, respectively [8,9]. Increasing search interest about a particular disease represents the development of an epidemic and reflects limited knowledge [10]. Therefore, evidence for interest in mpox in China is still needed to provide a reference for information management related to the spread of infectious diseases that have become a subject of public interest [11]. Online search activity reflects matters of concern in a population, and this activity is commonly captured by internet search engines, such as the Baidu index in China [12-14]. We used the Baidu index in this study to determine trends in online search activity related to mpox and changes in this activity after 4 key cutoff time points (the first imported mpox case in Taiwan, the announcement of a PHEIC, the first imported mpox case in Hong Kong, and the first imported mpox case in Chongqing) using a time-series interrupted analysis to obtain evidence for comprehensive information management at the geographical level. In addition, online search activity may vary across the 31 provinces, municipalities, and autonomous regions in China because of differences in socioeconomic development and other possible factors, including demographic characteristics, human health resources, and disease burden [12]. Therefore, we calculated a concentration index of online search activity and its changes, analyzed factors influencing changes in this index to observe economic disparities, and provide the resulting detailed evidence for public information management.

## Methods

### Online Search Activity

The Baidu index was used to retrieve data on internet search activity related to mpox. Baidu has been used to retrieve information on the interests of more than 75% of the Chinese population [15]. Data are obtained by calculating the number of searches for specific keywords [16]. Here, we extracted daily online search activity from May 19 to September 19, 2022, for the keyword *mpox*. Considering the different population sizes in the 31 provinces, municipalities, and autonomous regions, and in order to compare search volumes at a geographical level, we calculated the online search activity per 10,000 people.

### Covariates

Data on daily cumulative confirmed mpox cases was retrieved from Our World in Data [17]. Covariates were extracted from the China Health Statistics Yearbook 2022 [18] and included the total population size; life expectancy; gross domestic product (GDP) per capita; the number of medical and health institutions, health personnel, and hospital beds; total health expenditures; the number of outpatient services; AIDS incidence per 100,000 people; AIDS mortality per 100,000 people; gonorrhea incidence per 100,000 people; and syphilis incidence per 100,000 people.

### Statistical Analysis

We used interrupted time-series analysis (ITS) to estimate changes in online search activity after adjusting for potential seasonality, autocorrelation, and covariates. The 4 cutoff time points were all in 2022 and included June 24 (first imported mpox case in Taiwan), July 23 (announcement of a PHEIC), September 6 (first imported mpox case in Hong Kong) and September 16 (first imported mpox case in Chongqing ). Then, we analyzed the relationships between changes in online search activity and demographic characteristics, human health resources, and disease burden to explore influencing factors using a general linear regression (GLM) model.

In this study, the distribution of online search activity at an economic level was examined with the GDP per capita of each province. The Lorenz curve was obtained by plotting the cumulative percentage of daily online search activity per 10,000 people (on the y-axis) against the cumulative percentage of the population ranked by GDP per capita (on the x-axis). The concentration index (C) can be calculated using the following formula: [19]


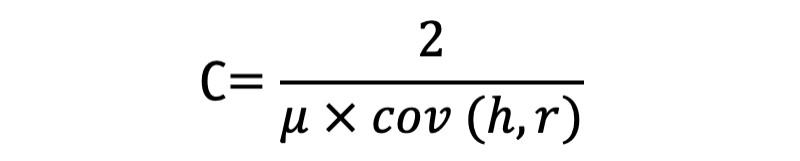


where h refers to the outcome, μ is the mean of h, and r denotes the fractional rank of individuals in the distribution used (economic status). The concentration index ranges between –1 and +1. A value of zero for concentration index represented absolute fairness, with no economic inequality. If the concentration index has a negative value, it indicates that online search activity was more concentrated among poor people (ie, pro-rich). Conversely, if the concentration index has a positive value, it indicates that online search activity was more concentrated among rich people (ie, pro-poor) [20]. All analyses were conducted in R (version 4.1.0; R Core Team).

### Ethical Approval

Ethical approval is not required as the deidentified data are publicly available.

## Results

### Trends in Online Search Activity at National and Provincial Levels

**Figure 1:**
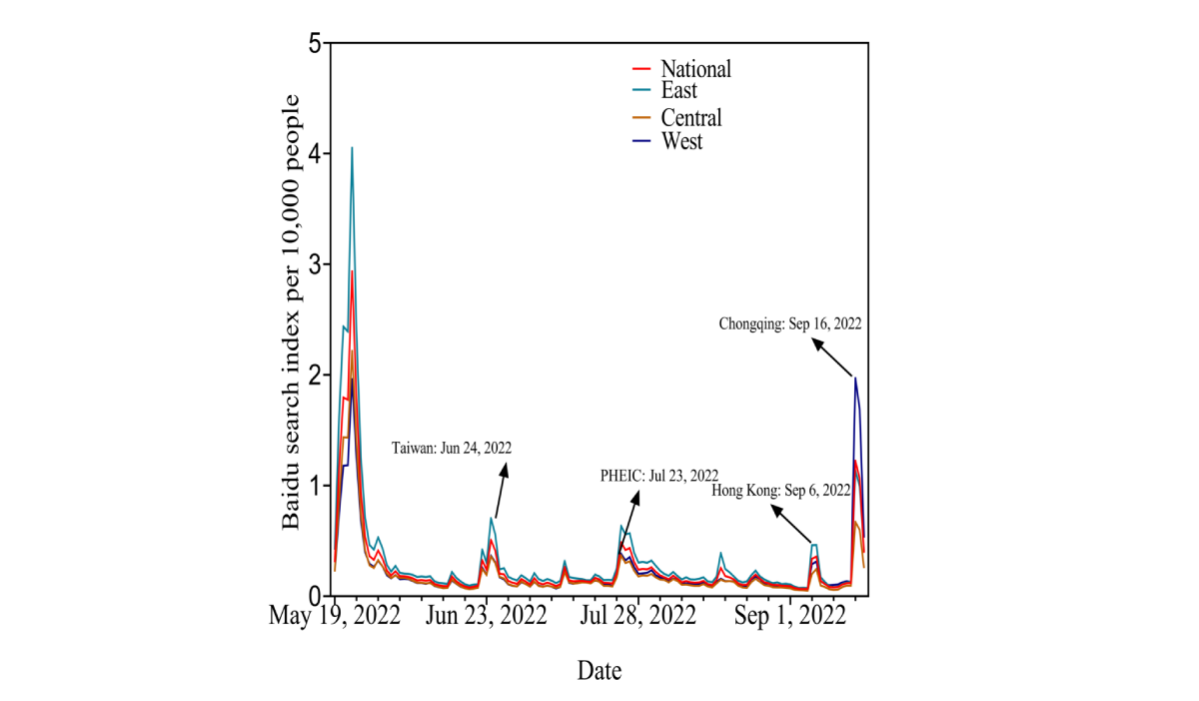
Daily online search activity related to mpox at national and regional levels in China from May 19 to September 19, 2022. PHEIC: public health emergency of international concern.

**Figure 2:**
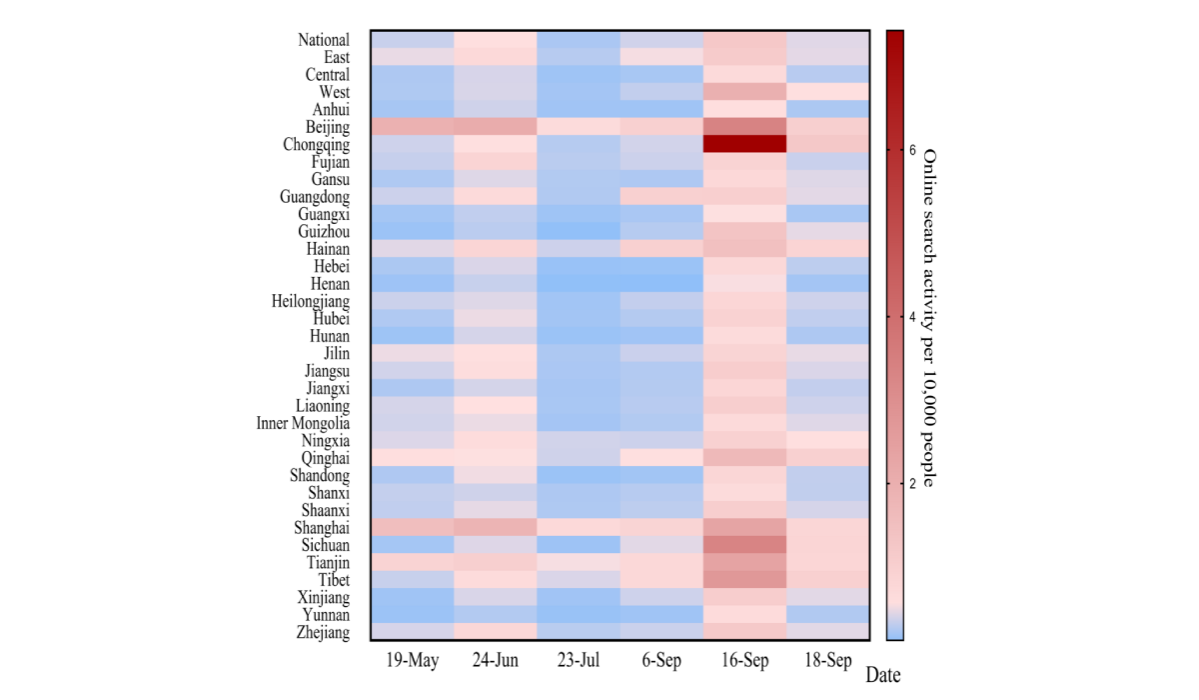
Heat plot of online search activity in 31 provinces, municipalities, and autonomous regions in China from May 19 to September 19, 2022. Redder shading indicates higher online search activity.

From May 9 to September 19, 2022, except for a peak in the early period, national online search activity peaked on June 24, July 23, September 6, and September 16, 2022 (September 16 saw the first imported mpox case in Chongqing and had an especially strong peak). There were similar patterns in the eastern, central, and western regions (Figure 1). Across the 31 provinces, municipalities, and autonomous regions in the early period, Beijing and Shanghai were the top 2 areas with higher online search activity. Online search activity was clearly highest on the day of the first imported mpox case in Chongqing when compared to the other 3 cutoff time points (Figure 2).

**Figure 3:**
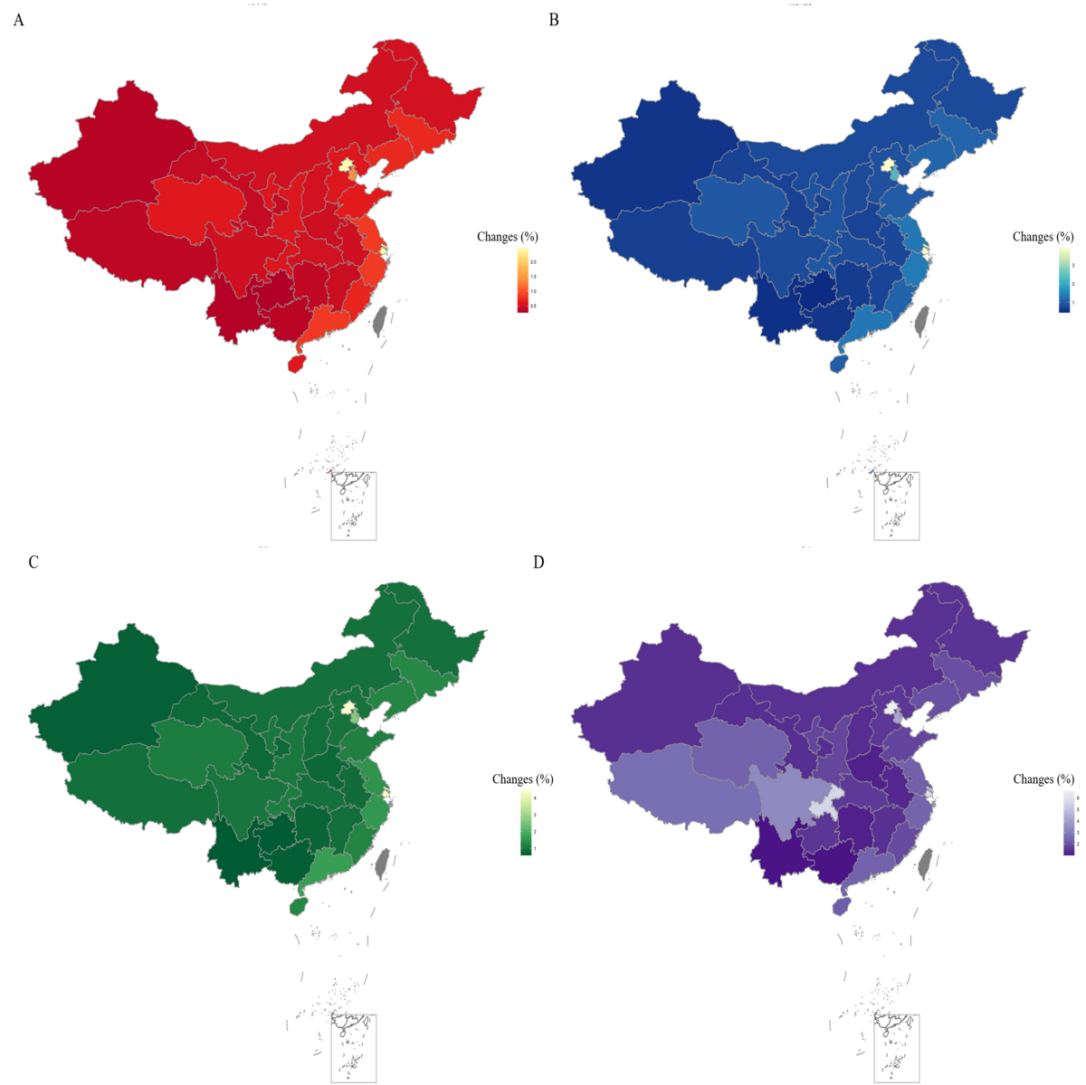
Changes in online search activity after 4 cutoff time points in 31 provinces, municipalities, and autonomous regions of China from May 19 to September 19, 2022. (A) Change in online search activity after the first imported mpox case in Taiwan; (B) change in online search activity after the announcement of the public health emergency of international concern; (C) change in online search activity after the first imported mpox case in Hong Kong; (D) change in online search activity after the first imported mpox case in Chongqing. Lighter colors indicate greater changes.

After the day of the first imported mpox case in Taiwan, announcement of the PHEIC, the first imported mpox case in Hong Kong, and the first imported mpox case in Chongqing, national online search activity increased by 0.642%, 1.035%, 1.199%, and 2.023%, respectively (Table 1). The increases were higher at all time points in the eastern regions compared to the central and western regions (Table 1). Across the 31 provinces, municipalities, and autonomous regions, the 3 provinces with the highest increases were Beijing, Shanghai, and Tianjin, until the day of the first imported mpox case in Chongqing, after which Chongqing replaced Tianjin (Table 1 and Figure 3).

**Table 1:** Changes in online search activity related to mpox in China and its 31 provinces, municipalities, and autonomous regions from May 19 to September 19, 2022.

Areas		First imported mpox case in Taiwan		Announcement of public health emergency of international concern		First imported mpox case in Hong Kong			First imported mpox case in Chongqing	
		Change, %	*P* value	Change, %	*P* value	Change, %	*P* value	Change, %		*P* value
National		0.642	<.001	1.035	<.001	1.199	<.001	2.023		<.001
**Regions**										
	East	0.886	<.001	1.417	<.001	1.633	<.001	2.408		<.001
	Central	0.487	<.001	0.777	<.001	0.887	<.001	1.351		<.001
	West	0.427	<.001	0.714	<.001	0.858	<.001	2.159		<.001
**Provinces, municipalities, and autonomous regions**										
	Anhui	0.486	<.001	0.781	<.001	0.875	<.001	1.283		<.001
	Beijing	2.483	<.001	3.943	<.001	4.440	<.001	6.648		<.001
	Chongqing	0.576	.01	0.966	.003	1.141	.009	5.586		<.001
	Fujian	0.696	<.001	1.093	<.001	1.247	<.001	1.854		<.001
	Gansu	0.419	<.001	0.715	<.001	0.831	<.001	1.373		<.001
	Guangdong	0.823	<.001	1.332	<.001	1.667	<.001	2.312		<.001
	Guangxi	0.340	<.001	0.574	<.001	0.676	<.001	1.014		<.001
	Guizhou	0.270	<.001	0.460	<.001	0.627	<.001	1.508		<.001
	Hainan	0.601	<.001	1.038	<.001	1.307	<.001	2.225		<.001
	Hebei	0.586	<.001	0.897	<.001	0.983	<.001	1.525		<.001
	Heilongjiang	0.515	<.001	0.816	<.001	0.958	<.001	1.474		<.001
	Henan	0.471	<.001	0.730	<.001	0.815	<.001	1.176		<.001
	Hubei	0.509	<.001	0.831	<.001	0.952	<.001	1.565		<.001
	Hunan	0.399	<.001	0.650	<.001	0.745	<.001	1.183		<.001
	Inner Mongolia	0.495	<.001	0.816	<.001	0.945	<.001	1.419		<.001
	Jiangsu	0.840	<.001	1.334	<.001	1.505	<.001	2.265		<.001
	Jiangxi	0.460	<.001	0.751	<.001	0.899	<.001	1.407		<.001
	Jilin	0.720	<.001	1.115	<.001	1.295	<.001	1.910		<.001
	Liaoning	0.720	<.001	1.118	<.001	1.254	<.001	1.934		<.001
	Ningxia	0.578	<.001	0.917	<.001	1.046	<.001	1.754		<.001
	Qinghai	0.606	<.001	0.967	<.001	1.115	<.001	2.333		<.001
	Shaanxi	0.584	<.001	0.929	<.001	1.053	<.001	1.760		<.001
	Shandong	0.640	<.001	1.023	<.001	1.168	<.001	1.748		<.001
	Shanghai	2.400	<.001	3.931	<.001	4.297	<.001	5.902		<.001
	Shanxi	0.512	<.001	0.824	<.001	0.914	<.001	1.364		<.001
	Sichuan	0.487	.001	0.819	<.001	1.016	<.001	3.344		<.001
	Tianjin	1.425	<.001	2.302	<.001	2.670	<.001	4.172		<.001
	Tibet	0.366	.002	0.693	<.001	0.937	<.001	2.591		<.001
	Xinjiang	0.324	<.001	0.565	<.001	0.697	<.001	1.400		<.001
	Yunnan	0.291	<.001	0.503	<.001	0.595	<.001	1.018		<.001
	Zhejiang	0.841	<.001	1.368	<.001	1.566	<.001	2.380		<.001

Changes after the day of the first imported mpox in Chongqing were affected by the AIDS incidence per 100,000 people. When it increased by 1 per 100,000 people, changes increased by 36.22% (95% CI 3.29%-69.15%; *P*=.04) after controlling for other covariates.

### Economic Disparities in Online Search Activity and Changes in Activity

**Figure 4:**
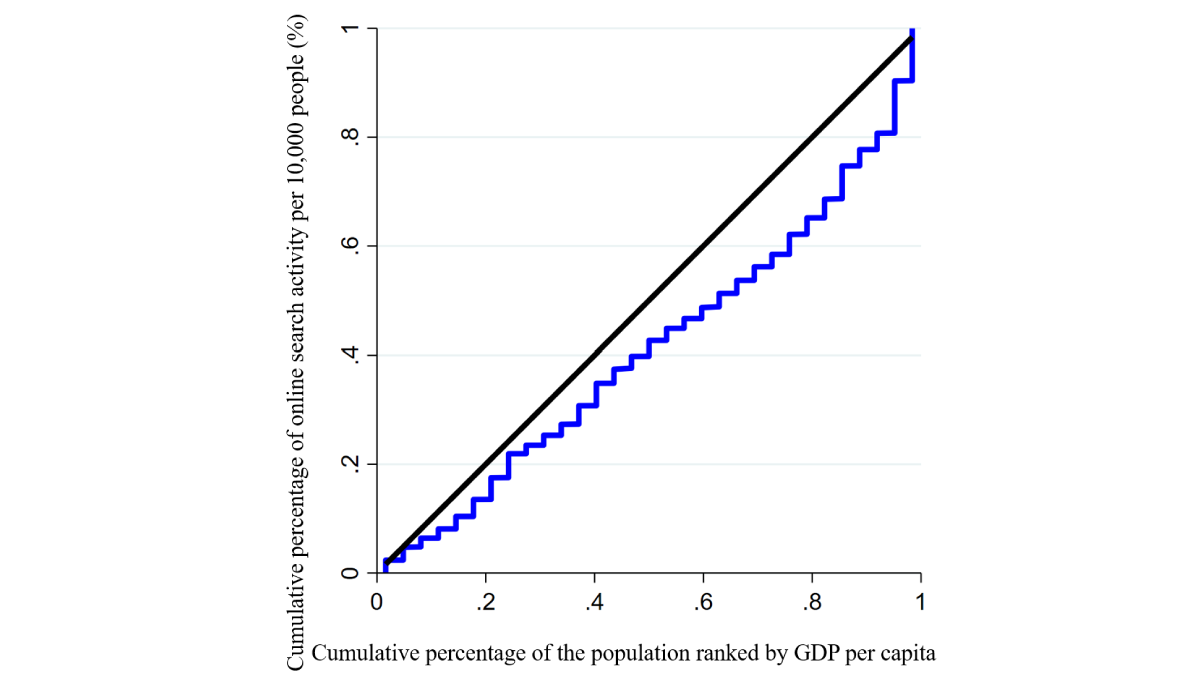
Lorenz curves for online search activity related to mpox in China from May 19 to September 19, 2022. The black line represents the equality line; the concentration index was 0.177 (*P*<.001). GDP: gross domestic product.

The Lorenz curves for online search activity are shown in Figure 4. The curves (concentration index=0.177; *P*<.001) lay below the equality line and had a positive value, indicating that economic-related disparities and inequality disadvantageous to the rich (ie, pro-poor) in online search activity were more concentrated among populations with a higher economic status. There was a significant difference between the western, eastern, and central areas (*P* for difference <.001). Unlike for the central area, the eastern area (concentration index=0.234; *P*<.001) and the western area (concentration index=0.047; *P*=.04) had significant economic-related disparities.

**Figure 5:**
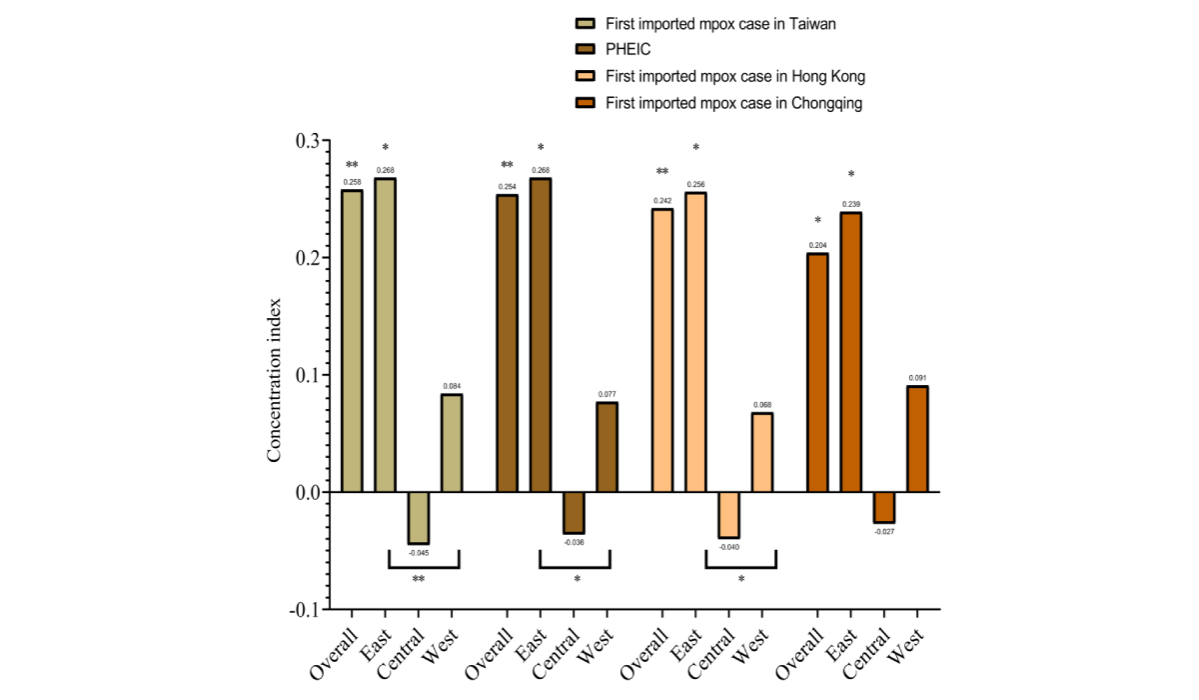
The concentration index for changes in online search activity related to mpox in China from May 19 to September 19, 2022.*P for difference <.01; **P for difference <.001.

The concentration index for changes in online search activity is shown in Figure 5. Changes in online search activity were also more concentrated among populations with a higher economic status. The overall concentration index for changes in online search activity became lower. The first 3 cutoff points had significant differences between the western, eastern, and central areas (all *P* for difference <.05), except for the day of the first imported mpox case in Chongqing (*P*=0.083).

## Discussion

### Principal Findings

To our knowledge, this is the first study to assess trends and influencing factors for online search activity related to mpox in China. Our study found that online search activity and changes in this activity were highest on the day of the first imported mpox case in Chongqing. The eastern area always had stronger changes than the central and western areas. Across 31 provinces, municipalities, and autonomous regions, the areas with the top 3 strongest changes were Beijing, Shanghai, and Tianjin, except for the day of the first imported mpox case in Chongqing, when Chongqing replaced Tianjin. Online search activity and changes in this activity were more concentrated among populations with a higher economic status, but the overall concentration index of changes in online search activity became lower over time. When AIDS incidence increased by 1 per 100, 000 people, the change after the day of the first imported mpox case in Chongqing increased by 36.22%.

Clearly, compared with the day of the first imported case in Taiwan, the announcement of the PHEIC, and the first imported mpox case in Hong Kong, online search activity and changes in this activity were highest on the day of the first imported mpox case in Chongqing. There is no doubt that every emergency increases people’s interest in mpox. Geographical distance may influence people’s interest. Therefore, people may have increased searching for the latest knowledge to reduce their risk of infection when the first imported mpox case was identified in Chongqing compared to when the first cases were identified in Taiwan and Hong Kong. China is a country with a broad land area and more than 1.4 billion people, creating difficulties for the prevention and control of infectious diseases. In order to prepare for prevention and control work, China announced the Mpox Diagnosis and Treatment Guidelines (2022 Edition) on June 10, 2022 [21], and the Frontier Health and Quarantine Law of the People’s Republic of China and other regulations on July 24, 2022 [22]. Our findings provide additional evidence to inform prevention and control measures. If people lack knowledge on the transmission of the virus and prevention measures, they seek information more frequently; meanwhile, the possibilities of finding inaccurate information are higher [23]. Therefore, interest in mpox reminds us that trusted sources should be developed to supplement access to reliable information in a timely manner.

Furthermore, we found that online search activity and changes in this activity were more concentrated among populations with a higher economic status. Populations in provinces with a higher economic status may pay more attention to infectious diseases. However, the overall concentration index of changes in online search activity became lower over time. When the first imported case was identified in Chongqing, nearly all provinces focused on mpox; thus, health education and trusted sources of supplemental reliable information became more urgent over time all across China, not only in higher economic status regions, but also in low-income areas.

Our study found that AIDS incidence was associated with changes in online search activity after the day of the first imported mpox case in Chongqing. When AIDS incidence increased by 1 per 100,000 people, online search activity increased by 36.22%. This finding indicates that at this moment, provinces with higher AIDS incidence have more interest in mpox. Due to the same population being at high risk for both mpox and AIDS—men who have sex with men (MSM)—the increase in online search activity was associated with higher AIDS incidence. Our study shows that strengthening health education for MSM in provinces with higher AIDS incidence is critical. COVID-19 and mpox have similar prodromes, including fever and sore throat, making correct online information and accurate public knowledge of mpox in China crucial [24,25].

Some limitations of this study should be mentioned. Firstly, Baidu is not the only internet search engine, so online search activity in this study may have been underestimated. However, as Baidu is used by more than 75% of the Chinese population, and our study mainly compares regional and economic differences in online search activity in China, this limitation had little impact on our results. Secondly, data on internet attention related to mpox was unavailable before 2022, so we could not compare trends in 2022 with other years. Finally, due to privacy protection, we lack detailed data about the ages and occupations of the population under study, which might have limited the depth of our analysis.

### Conclusion

Regions with a higher economic level showed more interest in mpox, especially Beijing and Shanghai. After the day of the first imported mpox case in Chongqing, changes in online search activity were affected by AIDS incidence, and economic-related disparities became lower over time. Constructing reliable information sources for higher-level economic regions and regions with a higher AIDS burden, as well as promoting public knowledge in lower-level economic regions, especially after important events that affect public opinion, is important in China.

## Abbreviations

GDP: gross domestic product
ITS: interrupted time-series analysis
MSM: men who have sex with men
PHEIC: public health emergency of international concern
WHO: World Health Organization

## Data Availability

The data sets analyzed during this study are available in the Baidu index [16] and Our World in Data [17].
